# Local application of rapamycin reduces epidural fibrosis after laminectomy via inhibiting fibroblast proliferation and prompting apoptosis

**DOI:** 10.1186/s13018-016-0391-0

**Published:** 2016-05-06

**Authors:** Yu Sun, Shuai Zhao, Xiaolei Li, Lianqi Yan, Jingcheng Wang, Daxin Wang, Hui Chen, Jihang Dai, Jun He

**Affiliations:** Department of Orthopedics, Clinical medical college of Yangzhou University, Nantong West Road 98, Yangzhou, Jiangsu 225001 China; Department of Orthopedics, Xiangya Second Hospital, Central South University, Changsha, Hunan 410012 China; Orthopedics Institute, Subei People’s Hospital of Jiangsu Province, Yangzhou, Jiangsu 225001 China

**Keywords:** Rapamycin, Epidural fibrosis, Laminectomy, Fibroblast, Apoptosis, Proliferation

## Abstract

**Background:**

Epidural fibrosis is a common complication after laminectomy. It is associated with intractable lower back pain and additional complications. To date, no study has evaluated whether the local application of rapamycin (RAPA) can inhibit fibroblast proliferation and reduce epidural scar adhesion after laminectomy. The results of the present study showed that the local application of RAPA reduces epidural fibrosis after laminectomy in rats.

**Methods:**

In this study, 32 male Sprague-Dawley rats were randomly divided into four groups (0.2 mg/ml RAPA-treated group, 0.1 mg/ml RAPA-treated group, 0.05 mg/ml RAPA-treated group and physiological saline group). Laminectomy was performed at the level of lumbar segment 1 to 2, and different concentrations of RAPA or saline were applied to the laminectomy sites for 10 min. Four weeks after laminectomy, the rats were sacrificed, and the degrees of epidural adhesion in each group were evaluated. Macroscopic assessment, analysis of hydroxyproline content, and histological analysis were used to determine the therapeutic effect of the local application of RAPA on the inhibition of fibroblast proliferation and the reduction of epidural fibrosis after laminectomy. Next, we cultured fibroblasts from epidural scar tissues of rats that had undergone laminectomy. Fibroblasts were exposed to the indicated concentrations of RAPA, and western blotting and TUNEL assays were used to assess the effects of RAPA on inhibiting fibroblasts proliferation and promoting fibroblast apoptosis.

**Results:**

The results of macroscopic assessments, analysis of hydroxyproline content, and histological analyses indicated that RAPA significantly inhibited fibroblast proliferation and reduced epidural fibrosis in the treated groups in the rat model. The western blotting results indicated that the expression levels of the pro-apoptotic proteins cleaved-PARP and Bax were up-regulated, whereas those of Bcl-2 were reduced. TUNEL assay indicated that the apoptosis rates of fibroblasts were significantly increased after exposure to the indicated concentrations of RAPA.

**Conclusions:**

The local application of RAPA reduced epidural fibrosis after laminectomy by inhibiting the proliferation of fibroblasts, stimulating their apoptosis, and decreasing collagen synthesis. This protocol may be used in new clinical treatment strategies to reduce epidural fibrosis after laminectomy.

## Background

Extensive epidural fibrosis is a troublesome complication after laminectomy. It can cause various clinical conditions, such as failed back pain surgery syndrome (FBSS), which is characterized by chronic radicular nerve or lower back pain [1–3]. These types of complications make revision surgeries more complex and time-consuming, and the results of reoperation are often poor [4, 5]. Therefore, a solution to this problem is greatly needed.

Many studies have explored ways to prevent epidural scar adhesion after laminectomy. Many types of biological and synthetic materials, such as polymethyl methacrylate, polylactic acid, autologous leather, silastic silicone, and fat grafts, have shown anti-fibrotic effects [6–10]. Pharmaceutical agents, such as mitomycin C, hydroxycamptothecine, colchicine, steroid hormone and anti-inflammatory agents, have been used to reduce epidural fibrosis [11–14]. However, limited or variable success has been achieved, and some medicines cause side effects such as wound infection. Therefore, it is still clinically urgent to explore new methods for reducing epidural fibrosis. Recently, it has been reported that macrolide antibiotics, such as azithromycin and tacrolimus (FK506), exhibit effectiveness in reducing the fibrosis of epidural scars [13, 15].

RAPA, also called sirolimus, is a type of macrolide antibiotic and immunosuppressive agent [16–18]. Animal studies and clinical trials have shown that it has low renal toxicity and good effectiveness. It is widely used to prevent transplantation rejection [19, 20]. Many studies have indicated that RAPA is effective in inhibiting scar adhesion. In ophthalmology, the local application of RAPA is used to prevent scar adhesion after cataract surgery [21–24]. Studies on pathological scars have indicated that RAPA inhibits the proliferation of fibroblasts [25–27]. However, no study has determined whether the local application of RAPA can reduce epidural fibrosis after laminectomy.

In the present study, we sought to evaluate the therapeutic effect of local application of RAPA on inhibiting fibroblast proliferation and reducing epidural fibrosis. The results of this study may provide a novel method to reduce the adhesion of epidural scars, which may be useful in future human trials for clinical applications.

## Methods

### Animals, cells and reagents

Thirty-two healthy male Sprague-Dawley rats (mean weight of 350 g) were purchased from Shanghai Laboratory Animal Center. The animals received care in compliance with the principles of International Laboratory Animal Care, and the experimental protocol was approved by the Animal Care and Research Committee of Yang Zhou University (Yangzhou, China). RAPA, which was purchased from Astellas Ireland Co, Ltd. (Killorhlin, Co. Kerry, Ireland), was dissolved in dimethyl sulfoxide (DMSO) purchased from Sigma-Aldrich (St Louis, MO, USA) and stored at −20 °C. The RAPA solution was diluted with saline or cell culture medium such that the DMSO comprised < 0.1%. Specific antibodies against cleaved-PARP, Bcl-2 and Bax were purchased from Cell Signaling Technology (Cambridge, MA, USA). All rats were randomly divided into four groups (eight rats in each group): 0.05 mg/ml RAPA, 0.1 mg/ml RAPA, 0.2 mg/ml RAPA, and saline groups. Before the experiment, the rats were housed for 1 week and allowed to adapt to the conditions of the laboratory.

A primary fibroblast cell line was cultured from the epidural scar tissue of rats that had undergone laminectomies. Briefly, the scar tissues at L1–L2 level were anatomically separated and rinsed immediately with phosphate-buffered saline, then cut into 1 × 1 mm^2^ sections and cultured in Dulbecco’s modified Eagle’s medium (DMEM; Invitrogen, CA, USA) supplemented with 10 % foetal bovine serum, 0.1 U/l penicillin, and 50 μg/ml streptomycin (Gibco, CA, USA) at 37 °C in 5 % CO_2_. Culture media were replaced every 2 days until fibroblasts reached approximately 80 % confluence. The cells of passages 3 to 6 were used in all experiments, the cells were transfered into a serum-free medium overnight and then subjected to various reagent treatments with varying doses of RAPA, as indicated in the figure legends.

All other reagents were purchased from Sigma Aldrich (St Louis, MO, USA) unless stated otherwise.

### Construction of the laminectomy model

A rat laminectomy model was used to determine the effects of RAPA on epidural fibrosis. Briefly, each rat was anaesthetized with an intraperitoneal injection of 1 % pentobarbital sodium solution (4 ml/kg body weight) and then fixed in the prone position. The animal’s back hair at the level of L1 and L2 was shaved, and skin was sterilized with iodophor three times. The laminectomy model was constructed according to methods described in previous studies [28, 29]. All procedures were performed under sterile conditions with basic surgical tools, surgical microscopes and an electrical drill. A median incision of the dorsal skin at the level of L1–L2 was made, and the paraspinal muscles were separated. A rongeur was used to remove the spinous process and vertebral plate, and the dura mater at the L1 to L2 level was exposed. Then, cotton soaked with different concentrations of RAPA (0.05 mg/ml, 0.1 mg/ml and 0.2 mg/ml) or saline was applied to the surgical site for 10 min (the volume of application was approximately 1 ml). The surrounding tissues were protected from contact with the agent. Satisfactory haemostasis was achieved by using gauze; bone wax and cauterization after laminectomy were not needed. All procedures were performed with care to avoid injury to the neural tissues.

### Macroscopic assessment of epidural fibrosis

Four weeks after the surgery, four rats in each group were randomly selected, and the surgical sites were reopened carefully. The degree of epidural fibrosis at the L1 to L2 level was evaluated by three professional blinded pathologists according to the Rydell classification [30] (grade 0 = epidural scar tissue was not adherent to the dura mater; grade 1 = epidural scar tissue was adherent to the dura mater but was easily dissected; grade 2 = epidural scar tissue was adherent to the dura mater and was difficult to dissect without disrupting the dura mater; and grade 3 = epidural scar tissue was firmly adherent to the dura mater and could not be dissected).

### Hydroxyproline content analysis

After macroscopic assessment of epidural fibrosis, the scar tissue (wet weight = approximately 4 mg) was collected from L1 to L2 area surrounding the laminectomy site. The content of hydroxyproline in the scar tissue of different groups was determined according to the methods described in previous study [31, 32]. Briefly, the samples were lyophilized, ground and hydrolysed with 6 mol/l HCl at 130 °C for 12 h separately. The samples were then neutralized with 2.5 N NaOH with methyl red as an indicator. Chloramine T (1 ml) was added to the hydrolysed samples along with four hydroxyproline standards of known concentrations. After incubation for 20 min at room temperature, the hydroxyproline developer was added to the samples and standards. The absorbance of the solution was measured at 558 nm with a spectrophotometer, and the hydroxyproline content per milligram of scar tissue was calculated according to the standard curve, which was constructed with serial concentrations of commercial hydroxyproline.

### Histological analysis

Four rats randomly selected from each group were sacrificed after 4 weeks for histological evaluation. The entire L1–L2 spine column, including the paraspinal muscles and epidural scar tissues, was collected and immersed in 10 % buffered formalin for 5 days. The specimens were decalcified in 10 % EDTA (Sigma Aldrich, St Louis, MO, USA) for 3 weeks and embedded in paraffin. Then, 4-mm transverse sections were made through the L1–L2 vertebra, and this was followed by staining with haematoxylin–eosin (HE) and Masson’s trichrome. Three counting areas (100 × 100 μm each) were selected at the middle and at the margins of the laminectomy sites, and the number of fibroblasts was calculated.

### Analysis of RAPA’s influence on the proliferation of fibroblasts

To further determine the effects of RAPA on the proliferation of fibroblasts, the fibroblasts were cultured in Dulbecco’s modified Eagle’s medium (DMEM; Invitrogen, CA, USA) supplemented with 10 % foetal bovine serum, 0.1 U/l penicillin, and 50 μg/ml streptomycin (Gibco, CA, USA) at 37 °C and 5 % CO_2_. Briefly, fibroblasts were plated in six-well plates at a density of 8 × 10^5^ cells/well and cultured in Dulbecco’s modified Eagle’s medium (DMEM; Invitrogen, CA, USA) supplemented with 10 % foetal bovine serum, 0.1 U/l penicillin, and 50 μg/ml streptomycin (Gibco, CA, USA) at 37 °C in 5 % CO_2_. After reaching approximately 80 % confluence, the cells were transferred into serum-free medium overnight and then subjected to treatment with different concentrations of RAPA (0, 0.01, 0.1, and 1 μg/ml) for 48 h, as indicated in the figure legends. Then, the number of fibroblasts and changes in cell morphology were observed under a light microscope.

### Western blotting analysis of apoptosis markers

To further determine the effect of RAPA on the expression of apoptotic proteins, such as cleaved-PARP, Bax, and Bcl-2, fibroblasts were seeded in six-well plates at a density of 5 × 10^5^ and cultured in DMEM supplemented with 10 % foetal bovine serum and 1 % penicillin. When the cells were confluent, they were transfered into serum-free medium overnight and then pre-treated with or without different concentrations of RAPA for 48 h. Then, total proteins were extracted from cultured cells using radioimmuno-precipitation assay (RIPA) lysis buffer (Sigma-Aldrich, St Louis, MO, USA). Protein concentrations were determined using a bicinchoninic acid assay (BCA; ThermoFisher, MA, USA). Thirty micrograms of each protein lysate was added to each lane and resolved using sodium dodecyl sulphate-polyacrylamide gel electrophoresis (SDS-PAGE, Sigma Aldrich, St Louis, MO, USA) on 12 or 6 % gels and transferred to polyvinylidene difluoride membranes (Millipore, Bedford, MA, USA). The primary antibodies were diluted in 1 % (*w*/*v*) skimmed milk powder in TBS-Tween and incubated overnight at 4 °C. Membranes were then washed and incubated with the appropriate secondary antibodies conjugated with IRDye 800CW (molecular weight = 1162 Da). Antibody reactivity was detected after exposure in an Odyssey infrared imaging system (LI-COR, Nebraska, USA).All the experiments were repeated three times.

### TUNEL assay in cultured fibroblasts

To evaluate the apoptotic rate of RAPA on fibroblasts, the cells were transferred into a serum-free medium overnight and then pre-treated with or without different concentrations of RAPA for 48 h, the apoptotic rate of fibroblasts was analysed using a TdT-mediated dUTP-biotin nick-end labelling (TUNEL) test system (Roche, USA) according to the manufacturer’s instructions. Following staining, the apoptotic features of cell death were examined via fluorescence microscopy. The obtained images were merged and analysed using Image J software. The percentage of TUNEL-positive cells was defined as the number of TUNEL-stained cells divided by the number of DAPI-stained cells. The cells were counted by a naive observer.

### Statistical analysis

The statistical analysis was performed with SPSS software (version 19.0). The results of the data were expressed as the means ± standard deviation. *P* values < 0.05 were considered statistically significant.

## Results

### Macroscopic assessment of epidural fibrosis

The surgery was well tolerated by all rats, without the occurrence of wound infection, neurological deficits or cerebrospinal leaks. The results of macroscopic observation indicated that soft or weak scar adhesion was found in the 0.2 mg/ml RAPA group. Moderate scar adhesion was seen in the 0.1 mg/ml RAPA group; the tissues could be dissected by manual traction with less bleeding. However, severe epidural adhesions were observed in both the 0.05 mg/ml RAPA group and control group; dissection of the scar tissue was difficult and was accompanied by bleeding and disruption of the dura mater. The levels of epidural fibrosis were evaluated according to the Rydell classification, and the results are shown in Table 1.

**Table 1:** The grade of epidural scar adhesion through macroscopic was evaluated in rats according to the Rydell standard

Group	Grade			
	0	1	2	3
Saline	0	0	0	4
0.05 mg/ml	0	0	1	3
0.1 mg/ml	0	3	1	0
0.2 mg/ml	3	1	0	0

Four rats were randomly selected from the RAPA-treated groups of different concentrations and saline group

### Histological analysis

**Fig. 1:**
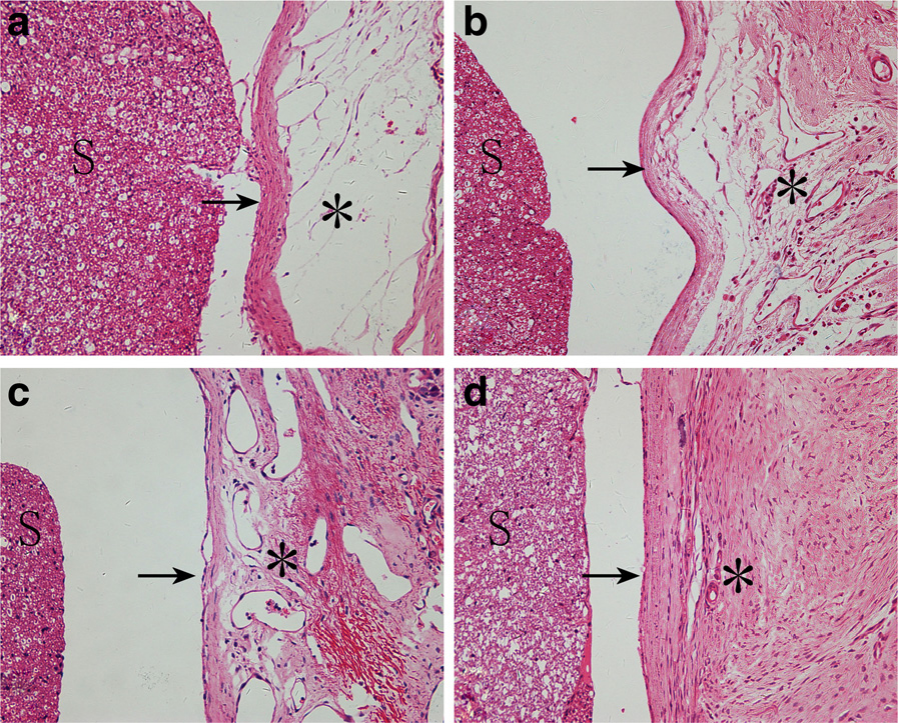
Photomicrographs of the epidural adhesions at the laminectomy sites from groups treated with 0.2 mg/ml RAPA (**a**), 0.1 mg/ml RAPA (**b**), 0.05 mg/ml RAPA (**c**) and the control group (**d**). Loose scar tissues (*asterisk*) with little adherence to the dura mater (*arrow*) were found in the 0.2 mg/ml RAPA group (**a**). Moderate scar tissues (*asterisk*) were found in the 0.1 mg/ml RAPA group (**b**). However, dense scar tissues (*asterisks*) adhered to the dura mater (*arrow*) were found in the 0.05 mg/ml RAPA (**c**) group and the control group (**d**). “S” represents the spinal cord. The sections were stained with haematoxylin–eosin. The magnification was ×200

**Fig. 2:**
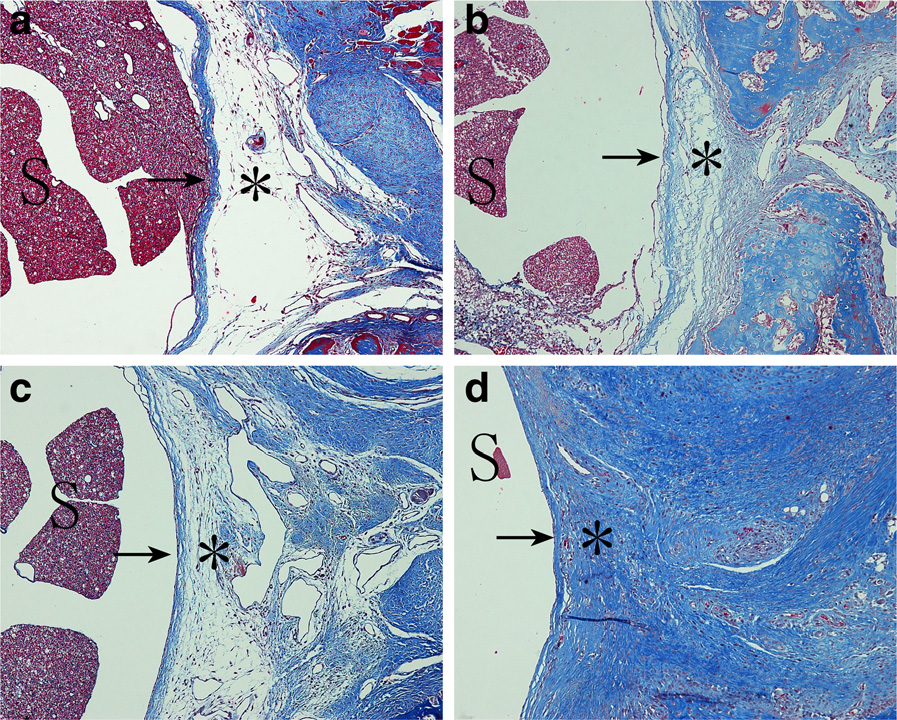
The effect of RAPA on collagen density in epidural adhesion issues in the laminectomy sites in groups treated with 0.2 mg/ml RAPA (**a**), 0.1 mg/ml RAPA (**b**), 0.05 mg/ml RAPA (**c**) or the control group (**d**). Collagen tissue is shown in *blue* because the sections are stained with Masson’s trichrome. The density of collagen in the sections from the 0.2 mg/ml RAPA-treated group (**a**) was clearly lower than that in tissue samples from the 0.1 mg/ml RAPA (**b**) and 0.05 mg/ml RAPA (**c**) groups and the control group (**d**). “S” represents the spinal cord. “*” represents the scar adhesion area of epidural. The sections were stained with masson. The magnification was ×100

The typical images of HE staining and masson staining of epidural scar tissues at L1–L2 levels were shown in Figs. 1 and 2, respectively. In both the control group and the 0.05 mg/ml RAPA group, dense epidural scar tissue and compact collagen tissues were found in the laminectomy sites, and the scar tissues were widely adhered to the dura mater and dorsal muscle (Figs. 1d and 2d; Figs. 1c and 2c). Moderate epidural scar adhesion and collagen tissues were found in the 0.1 mg/ml RAPA group (Figs. 1b and 2b). However, loose scar adhesion and collagen tissues were formed in the 0.2 mg/ml RAPA group (Figs. 1a and 2a).

### Hydroxyproline content analysis

**Fig. 3:**
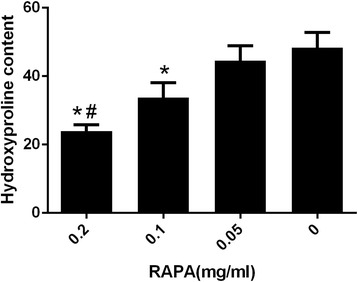
The effect of RAPA on the hydroxyproline content of epidural scar tissue in each group. Hydroxyproline levels (μg/mg) in epidural scar tissues of rats after treatment with different concentrations of RAPA and the control group were evaluated. *p < 0.05 versus the control group. #*p* < 0.05 in the 0.2 mg/ml RAPA group compared to the other RAPA groups

The hydroxyproline content of epidural scar tissue is shown in Fig. 3. The hydroxyproline content of the 0.2 mg/ml RAPA group was 33.08 ± 2.23 μg/mg, which was significantly lower than those of the 0.1 mg/ml RAPA group (43.39 ± 2.01 μg/mg), the 0.05 mg/ml RAPA group (55.08 ± 1.41 μg/mg) and the control group (58.14 ± 3.19 μg/mg) (#*p* < 0.05). The hydroxyproline content of the 0.1 mg/ml RAPA group was also lower than those of the 0.05 mg/ml RAPA group and the control group (*p < 0.05). However, there was no significant difference between the 0.05 mg/ml RAPA group and the control group (*p* = 0.203).

### Effect of RAPA on fibroblasts in epidural scar tissues

**Fig. 4:**
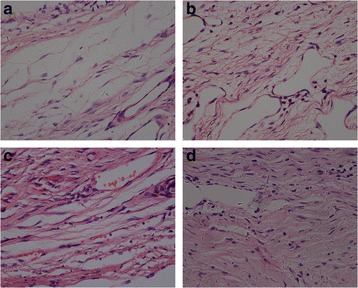
Histological observation of fibroblasts in the epidural scar tissue of the laminectomy defect areas in groups treated with RAPA of 0.2 mg/ml (**a**), 0.1 mg/ml (**b**), 0.05 mg/ml (**c**) or the control group(**d**). The number of fibroblasts in the 0.2 mg/ml RAPA group and 0.1 mg/ml RAPA group was lower than in the control group, but there was no significant difference between the 0.05 mg/ml RAPA group and control group. The fibroblast count in the 0.2 mg/ml RAPA group was also lower than those of the other RAPA groups. The sections were stained with haematoxylin–eosin and the magnification is ×400

**Fig. 5:**
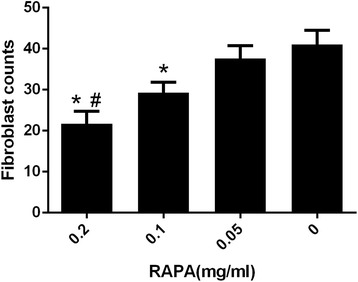
The numbers of fibroblasts in groups treated with different concentrations of RAPA (0.2 mg/ml, 0.1 mg/ml, 0.05 mg/ml) and the control group. *p < 0.05 compared to the control group. #*p* < 0.05 in the 0.2 mg/ml RAPA group compared to the 0.1 mg/ml RAPA group and 0.05 mg/ml RAPA group

The fibroblasts of epidural scar tissue and fibroblast counts in each group are shown in Figs. 4 and 5, respectively. The fibroblast counts were 19 ± 2.65 in the 0.2 mg/ml RAPA group, 34.67 ± 2.52 in the 0.1 mg/ml RAPA group, and 50.33 ± 2.08 in the 0.05 mg/ml RAPA group. The 0.2 mg/ml RAPA group and the 0.1 mg/ml RAPA group showed significantly lower fibroblast counts than the control group (54.67 ± 3.21) (*p < 0.05), but there was no significant difference between the 0.05 mg/ml RAPA group and the control group (*p* = 0.122). The number of fibroblasts in the 0.2 mg/ml RAPA groups was also significantly lower than those of the other RAPA groups (#*p* < 0.05).

### Effects of RAPA on the proliferation and apoptosis of fibroblasts

**Fig. 6:**
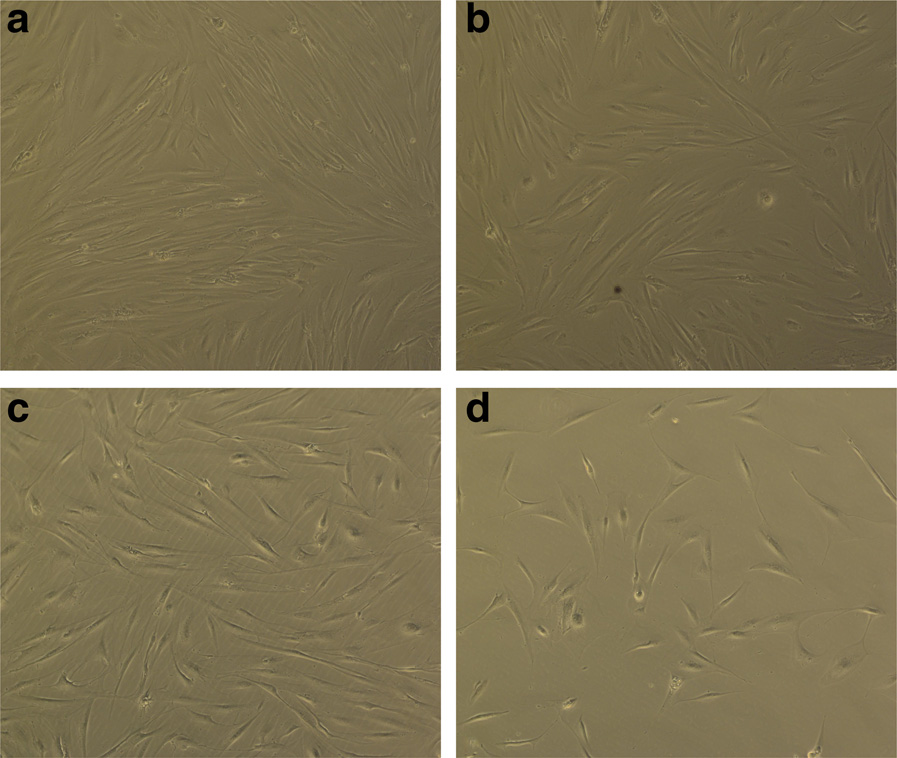
After fibroblasts were cultured with different concentrations of RAPA (0.01 μg/ml (**b**), 0.1 μg/ml (**c**), 1 μg/ml (**d**)) or control (**a**) for 48 h, the number of cells and cellular morphology were observed under a light microscope. The magnification was ×400

As shown in Fig. 6, after fibroblasts were transfered into serum-free medium overnight and treated with different concentrations of RAPA (0, 0.01, 0.1, and 1 μg/ml) for 48 h, the proliferation of fibroblasts was clearly inhibited. The number of fibroblasts decreased with increasing doses of RAPA.

**Fig. 7:**
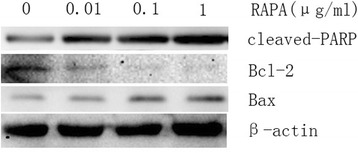
The expression levels of cleaved-PARP, Bax, and Bcl-2 in fibroblasts after treatment with different concentrations of RAPA (0.01 μg/ml, 0.1 μg/ml, and 1 μg/ml) for 48 h. When the dose of RAPA increased, the levels of the pro-apoptotic proteins cleaved-PARP and Bax increased; however, the levels of the anti-apoptotic protein Bcl-2 decreased

To evaluate the effect of RAPA on the apoptosis of fibroblasts, western blotting was used to determine the expression of the pro-apoptotic proteins cleaved-PARP and Bax and the anti-apoptotic protein Bcl-2 after fibroblasts were treated with different concentrations of RAPA. With increasing doses of RAPA, the expression levels of cleaved-PARP and Bax increased. However, the expression level of the anti-apoptotic protein Bcl-2 decreased. The results are shown in Fig. 7.

**Fig. 8:**
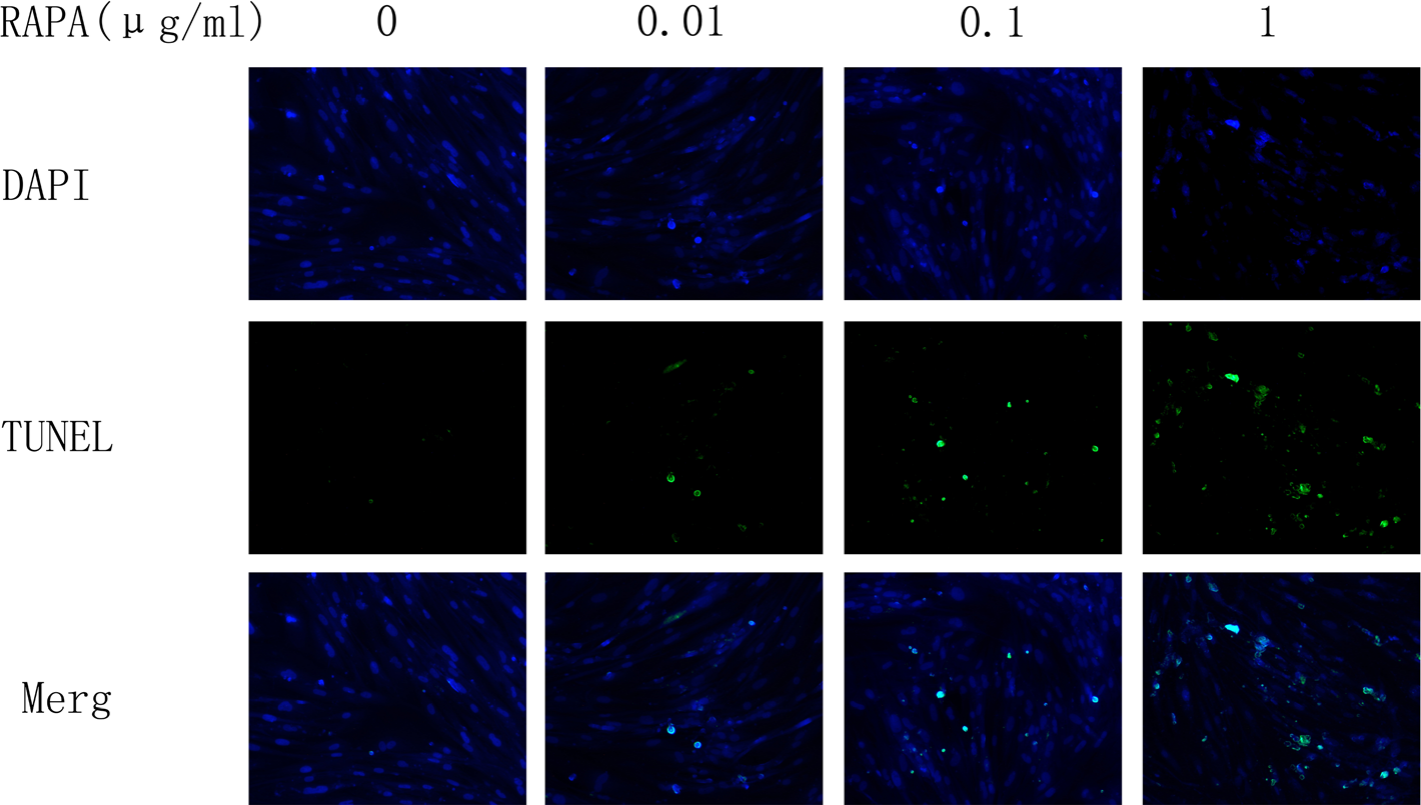
The results of TUNEL staining after fibroblasts were treated with different concentrations of RAPA (0.01 μg/ml, 0.1 μg/ml, and 1 μg/ml) for 48 h are shown. The nuclei of fibroblasts were stained with DAPI. After TUNEL staining, the nuclei of apoptotic fibroblasts were green. When the dose of RAPA increased, the number of apoptotic nuclei increased. The images were observed using fluorescence microscopy. The magnification is ×400

**Fig. 9:**
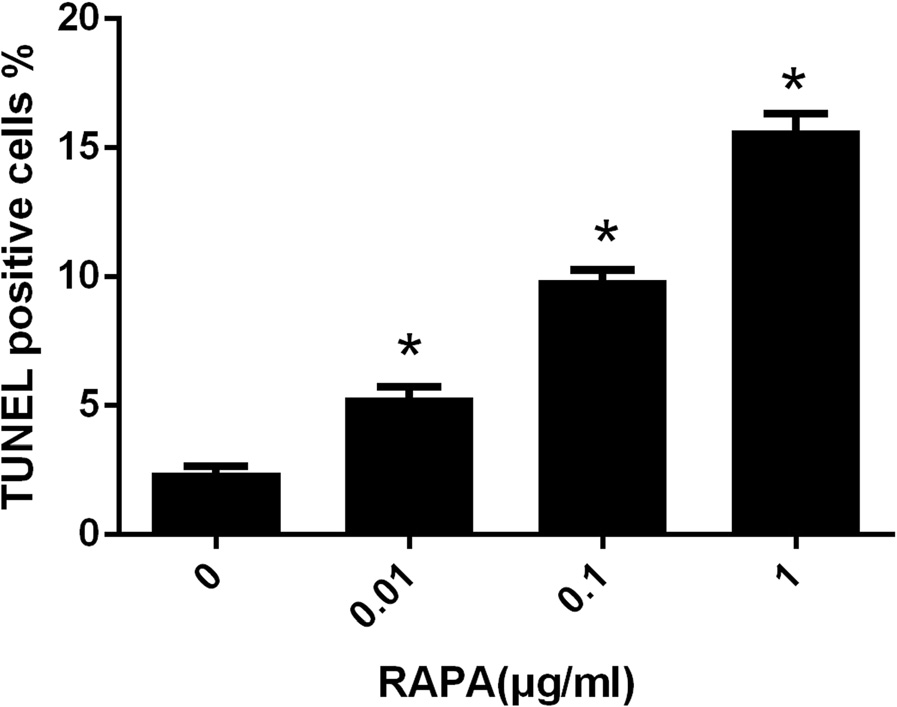
The percentages of TUNEL-positive cells in each group. Three fields were counted to obtain the percentages of TUNEL-positive cells in each group. *p < 0.05 compared with the control group

As shown in Fig. 8, the results of the TUNEL assay indicated that few apoptotic fibroblasts were found in the control group. After fibroblasts were treated with different concentrations of RAPA, the number of apoptotic fibroblasts increased. As shown in Fig. 9, compared with those of the control group, the rates of TUNEL-positive cells increased significantly in the fibroblasts of RAPA-treated groups (*p < 0.05).

## Discussion

Currently, the exact mechanisms that underlie the development of epidural fibrosis after laminectomy are still uncertain. It has been found that immune and inflammatory reactions play key roles in the formation of epidural fibrosis [33, 34]. After being stimulated by various immune cytokines, fibroblasts tend to proliferate extensively, produce collagen, and release extracellular matrix, which ultimately results in the formation of epidural scar adhesion. Excessive scar tissues tend to adhere to the dura mater or nerve roots in the vertebral canal, thus resulting in nerve root entrapment and limited nerve root mobility. Therefore, pharmaceutical agents that inhibit immune and inflammatory reactions and thus inhibit the proliferation of fibroblasts may be effective in reducing epidural scar adhesion.

RAPA is a new type of immunosuppressive drug characterized by a better clinical effect, low toxicity, and no renal toxicity (Fig. 9). Currently, RAPA is widely used to prevent the acute rejection of allograft transplants and suppress tumour growth [35, 36]. Many studies have shown that RAPA has anti-fibrosis properties. It has been found that 0.01 to 1 μg/ml RAPA inhibits the expression of MMP-1 and collagen in fibroblasts [37]. RAPA also suppresses the proliferation of fibroblasts. Additionally, the placement of a biodegradable rapamycin-eluting nano-fibre membrane-covered metal stent reduces fibroblast proliferation in experimental strictures in a canine model [38].

In this study, we cultured fibroblasts from the epidural scar tissues of rats after laminectomy and exposed the cells to various concentrations of RAPA. After fibroblasts were cultured with different concentrations of RAPA (0, 0.01, 0.1, and 1 μg/ml) for 48 h, the proliferation of fibroblasts was markedly inhibited. The number of fibroblasts decreased with increases in the concentration of RAPA. The western blotting results indicated that RAPA promoted the expression of pro-apoptotic proteins, such as Bax and cleaved-PARP, whereas it inhibited the expression of the anti-apoptotic protein Bcl-2 in fibroblasts. The results of the TUNEL assay indicated that RAPA promoted the apoptosis of fibroblasts.

Next, we constructed a laminectomy model in rats and treated them with different concentrations of RAPA, chosen on the basis of previous studies [37, 39]. We found that the local application of RAPA inhibited fibroblast proliferation in epidural scar tissues and reduced epidural fibrosis in rats. We evaluated the effect of RAPA in reducing epidural fibrosis by using several methods, including macroscopic assessment of epidural fibrosis, evaluation of hydroxyproline content, histological analysis, and fibroblast counting. The results indicated that the local application of RAPA inhibited fibroblast proliferation and reduced epidural fibrosis in a rat laminectomy model without any signs of wound infection, neurological deficits or cerebrospinal leakage. Additionally, 0.2 mg/ml RAPA exhibited better effectiveness than did lower concentrations of RAPA. Different concentrations of RAPA reduced epidural adhesion in a dose-dependent manner, except for the 0.05 mg/ml RAPA group, which showed no difference compared with the control group.

The results of this and previous studies show that RAPA can reduce epidural scar adhesion. RAPA prevents the progression of the cell cycle from G1 to S phases in T-lymphocytes and other cell types through various signal transduction pathways. It also inhibits the proliferation of immune cells, including fibroblasts. RAPA binds to immunophilin FKBP12, which blocks the nuclear factor of activated T-cells from entering the nucleus, reduces the production of cytokines such as IL-2 and alleviates the immune reaction [40]. As the number of inflammatory cytokines decreases, the number of fibroblasts decreases. Additionally, RAPA inhibits the expression of Bcl-2 in T-lymphocytes and other cell types, including fibroblasts; thus, RAPA promotes fibroblast apoptosis and accordingly decreases epidural fibrosis after laminectomy [41].

Considering these results, we concluded that the local application of RAPA may be an innovative and safe treatment for reducing epidural fibrosis. To accomplish this effect, RAPA affects the activity of fibroblasts after the immune reaction is alleviated. The present study is a preliminary assessment of the effect of RAPA on decreasing epidural fibrosis, using histological and histomorphometric analysis. A higher dosage of RAPA may produce better results, and more in-depth research on its mechanisms is still needed in the future.

## Conclusions

The local application of RAPA can reduce epidural fibrosis after laminectomy by inhibiting the proliferation of fibroblasts, stimulating their apoptosis, and decreasing collagen synthesis. This protocol may be used in new clinical treatment strategies to reduce epidural fibrosis after laminectomy.

### Ethics approval

All animals received care according to the principles of Laboratory Animal Care and international recommendations, and the experimental protocol was approved by the Animal Care and Research Committee of Central South University, China.

## Abbreviations

HE: haematoxylin and eosin
RAPA: rapamycin
